# Leptospiral outer membrane protein LipL32 induces inflammation and kidney injury in zebrafish larvae

**DOI:** 10.1038/srep27838

**Published:** 2016-06-09

**Authors:** Ming-Yang Chang, Yi-Chuan Cheng, Shen-Hsing Hsu, Tsu-Lin Ma, Li-Fang Chou, Hsiang-Hao Hsu, Ya-Chung Tian, Yung-Chang Chen, Yuh-Ju Sun, Cheng-Chieh Hung, Rong-Long Pan, Chih-Wei Yang

**Affiliations:** 1 Kidney Research Center and Department of Nephrology, Chang Gung Memorial Hospital, Chang Gung University College of Medicine, Taoyuan, Taiwan; 2 Department of Biochemistry and Molecular Biology, Chang Gung University College of Medicine, Taoyuan, Taiwan; 3 Department of Life Science and Institute of Bioinformatics and Structural Biology, College of Life Science, National Tsing Hua University, Hsinchu, Taiwan

## Abstract

Leptospirosis is an often overlooked cause of acute kidney injury that can lead to multiple organ failure and even death. The principle protein that conserved in many pathogenic leptospires is the outer membrane protein LipL32. However, the role of LipL32 in the pathogenesis of renal injury in leptospirosis is not entirely clear. Here we studied the effects of LipL32 on the developing kidney in zebrafish larvae. Incubation of zebrafish larvae with *Leptospira santarosai* serovar Shermani induced acute tubular injury predominantly in the proximal pronephric ducts. Furthermore, microinjection of *lipl32* mRNA or recombinant LipL32 protein into zebrafish larvae increased macrophage accumulation and disrupted the basolateral location of NA-K-ATPase in pronephric ducts. These changes led to substantial impairment of the pronephric kidney structure. We further demonstrated that morpholino knockdown of *tlr2*, but not *tlr4*, reduced the LipL32-induced leukocyte infiltration and kidney injury. These data demonstrate that LipL32 contributes to the renal pathology in leptospirosis and gives some clues to the potential virulence of LipL32. Our results support the use of zebrafish as a model organism for studying the disease mechanism of leptospiral infection. This model might permit the future exploration of the virulence and molecular pathways of different leptospiral outer membrane proteins.

Leptospirosis is an often overlooked cause of acute kidney injury that can lead to multiple organ failure and even death. It was estimated to cause one million cases worldwide yearly and the mortality rates were 5 to 10%^1^. The disease is a common zoonosis caused by exposure to *Leptospira* species, a family of spiral-shaped gram-negative spirochetes, in water or soil that are contaminated by the infected animals^2^,^3^,^4^. Sharing of *Leptospira* species pathogens between rodents, cattle, and fish in the same locality has been reported^5^. Pathogenic *Leptospira* species disseminates hematogenously and colonizes in renal proximal tubule epithelial cells^6^. The clinical manifestations of leptospirosis include fever, headache, muscle pain, jaundice, pulmonary hemorrhage, acute tubulointerstitial nephritis, and multiple organ failures^7^. Electrolyte imbalance such as sodium and potassium wasting is common in affected patients due to predominantly proximal tubular dysfunction^8^. Early diagnosis and prompt treatment with appropriate antibiotics are imperative to reverse the complications of leptospirosis^7^.

The candidate virulence factors for leptospiral infection include lipopolysaccharides (LPS)^9^, glycolipoproteins (GLP)^10^,^11^, hemolysins^12^, adhesion molecules^13^, and outer membrane proteins (OMPs)^14^,^15^, but their precise functions are still not completely understood. Among these factors, a 32-kDa lipoprotein, designated LipL32, is the most prominent protein in the leptospiral protein profiles and is highly conserved among pathogenic *Leptospira* species^16^. Anti-LipL32 reactivity was detectable in sera from leptospirosis patients in both acute and convalescent phases^17^. LipL32 has been shown to induce inflammatory cytokines and stimulates extracellular matrix production in cultured renal tubular epithelial cells through the TGF-beta1/Smad-dependent pathway^6^,^18^. These inflammatory reactions require Toll-like receptor 2 (TLR2) rather than TLR4 in cultured renal tubular cells^19^,^20^. Structurally, LipL32 contains the calcium-bound dock which is crucial for its interaction to host cells^21^,^22^. LipL32 recognizes and adheres to the individual components of extracellular matrix (ECM) including laminin, collagen I, and collagen V of the host cell^23^,^24^. However, the *in vivo* role of LipL32 in the pathogenesis of renal injury remains to be determined.

Zebrafish (*Danio rerio*) has been a vertebrate model for studying infectious diseases such as *Streptococcus, Salmonella*, and *Candida albican* infection^25^,^26^,^27^. These studies have demonstrated novel disease mechanisms that are not likely seen in other animal models. The advantages of zebrafish models include the ease of genetic manipulation, the ability to keep in germ-free conditions, and the resemblance of physiology to that of mammals^28^,^29^. There are only sparse data available on the use of zebrafish larvae for leptospirosis studies. An earlier study showed that microinjection of *L. interrogans* serovar Copenhageni stably infect zebrafish embryos^30^. The accumulation of macrophages surrounding the pronephric region suggests a role of leptospiral infection in causing kidney damage. However, the effects of leptospiral infection on the pronephric kidneys of zebrafish remain uninvestigated.

In this study, we have utilized a zebrafish model to study the effects of leptospiral infection and its major outer membrane protein, LipL32, on the developing kidneys. We demonstrated that LipL32 induced inflammation, reversed the polarity of NA-K-ATPase, and resulted in kidney injury. In addition, we found that LipL32 induced inflammation through TLR2 but not TLR4 by specifically knocking down these receptors in zebrafish larvae. These results may improve our understanding of the cellular and molecular mechanisms of the pathogenesis of leptospirosis.

## Results

### *Leptospira* infection caused tubular injuries in zebrafish larvae

**Figure 1:**
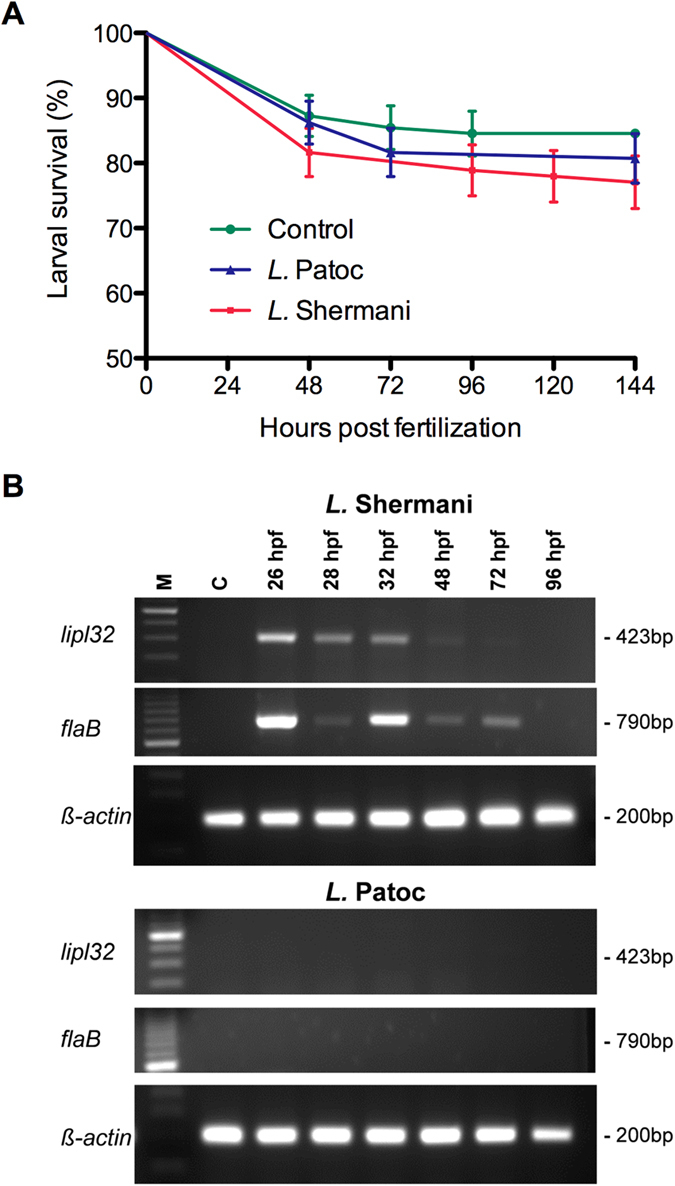
*Leptospira* Shermani infection in zebrafish larvae. (**A**) Kaplan-Meier survival curves of zebrafish larvae incubated with *L.* Shermani (n = 109), *L.* Patoc (n = 109) and controls (n = 110). Log-rank test *P* = 0.4248. Standard error was calculated by the Greenwood method. (**B**) RT-PCR analyses show the expression of *lipl32* and *flaB* mRNAs in zebrafish larvae incubated with *L.* Shermani but not with *L.* Patoc or E3 buffer. Zebrafish *β-actin* was used as a loading control. Cropped gel images are shown and the gels were run under the same experimental conditions. Uncropped gels are shown in Supplementary Figure S1.

To examine the virulence of *Leptospira* infection in zebrafish larvae, we first immersed zebrafish larvae to *L.* Shermani and *L.* Patoc from 24 hpf for 5 days (Fig. 1A). *L.* Shermani is the most frequently isolated pathogenic serovar in Taiwan and *L.* Patoc is a non-pathogenic leptospira^2^. The survival of zebrafish larvae treated with *L.* Shermani was 78.0% (85/109), whereas the survival of those treated with *L.* Patoc and E3 buffer control was 81.7% (89/109) and 85.3% (93/110), respectively. There was no significant difference in survival between groups (log-rank test, *P* = 0.4248). These results suggest that incubating zebrafish larvae with *L.* Shermani at a concentration of up to 1 × 10^6^ CFU/ml had no significant effects on larva viability.

To confirm the actual infection of zebrafish larvae with L. *Shermani*, we then investigated the expression of LipL32 in these larvae using Reverse Transcriptase PCR (RT-PCR). As shown in Fig. 1B, *lipl32* mRNA was detected in the zebrafish larvae that were incubated with *L.* Shermani but not in those treated with *L.* Patoc. The expression of *lipl32* mRNA appeared as early as two hours after incubation (26 hpf) and persisted until 48 hpf. We also examined the flagellin gene *flaB* mRNA, which showed a similar expression pattern but persisted until 72 hpf (Fig. 1B and Supplementary Figure S1)^31^. These results indicate a successful infection of *L.* Shermani in the zebrafish larvae using the immersion method.

**Figure 2:**
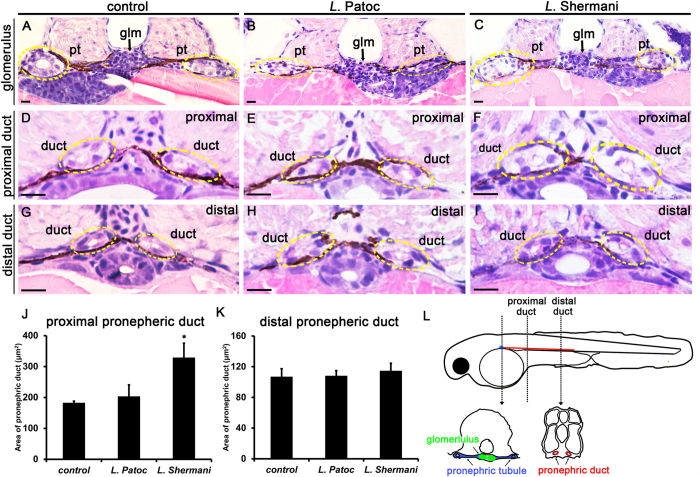
*Leptospira* Shermani induced acute tubular injury in zebrafish larvae. Transverse histological sections (H&E stain) from control (**A,D,G**), *L.* Patoc-treated (**B,E,H**), and *L.* Shermani-treated larvae (**C,F,I**). Note the marked swelling of the proximal pronephric ducts (circles) in *L.* Shermani-treated larvae compared with *L.* Patoc-treated larvae and controls. The differences were not evident in the distal pronephric ducts. Living larvae were incubated in E3 media containing *L*. Shermani or *L.* Patoc (1 × 10^6^ CFU/ml) from 24 hpf to 48 hpf. Control larvae were incubated with E3 buffer only. Representative micrographs from the level of the glomerulus (**A–C**), proximal pronephric duct (**D–F**), and distal pronephric duct (**G–I**) are shown. Circles indicate the location of the pronephric duct. Pt, pronephric tubule; Glm, glomerulus; Scale bar, 10 μm. (**J,K**) Quantification of the area of pronephric ducts. *P < 0.05 compared to control. n = 6 from three larvae in each group. (**L**) Diagram of transverse sections illustrating the structure of zebrafish pronephros at 48 hpf.

We then examined the pathological changes in pronephric kidneys after *L.* Shermani and *L.* Patoc infection. Compared with control larvae (Fig. 2A,D,G) and *L.* Patoc-infected larvae (Fig. 2B,E,H), the *L.* Shermani-infected larvae (Fig. 2C,F,I) showed marked swelling of the pronephric epithelial cells, predominantly in the proximal parts of pronephric ducts on histological examination. These results were confirmed by quantifying the area of pronephric ducts by image analysis on histology sections (Fig. 2J–L). Thus, our results demonstrated that infection with the pathogenic *L.* Shermani, which expresses the outer membrane protein LipL32, could cause acute tubular injury in zebrafish larvae. It seems unlikely that these changes are due to non-specific cytotoxicity because the larval survival was not jeopardized by current experimental conditions.

### Ectopic expression of *lipl32* mRNA in zebrafish larvae

**Figure 3:**
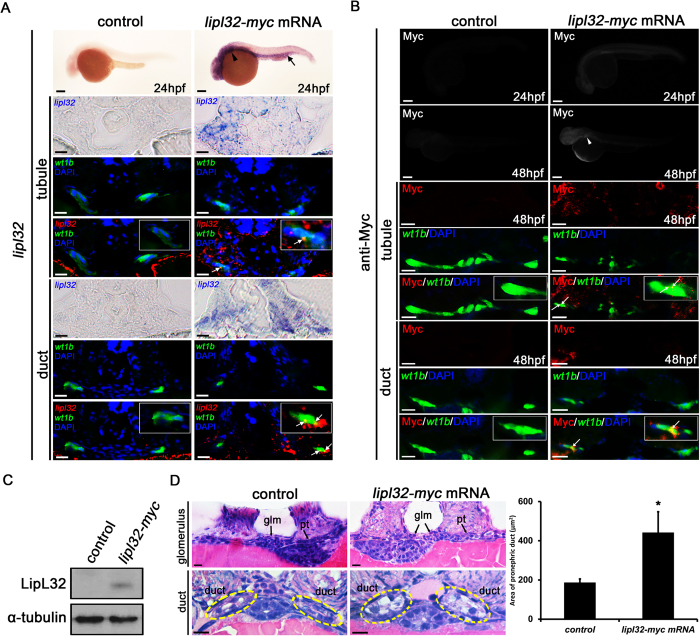
Microinjection of *myc*-tagged *lipl32* mRNA and ectopic expression of LipL32 in zebrafish larvae. (**A**) Whole-mount *in situ* hybridization for *lipl32* mRNA (lateral view, head to the left, scale bar, 200 μm). The expression of *lipl32* mRNA was prominent in the head, the pronephric region (arrowhead) and the posterior blood island (arrow) in the transgenic *wt1b*:GFP line at 24 hpf. In transverse sections, signals detected by *in situ* hybridization are pseudocolored in red and merged to GFP immunostaining (green) to locate the pronephros. The white arrows indicate colocalization (orange stain) of *lipl32* and GFP signals. Nuclei are stained with DAPI (blue). Scale bar, 20 μm. (**B**) Whole-mount immunostaining for Myc tag at 24 and 48 hpf. The expression of Myc tag was detected in the pronephric region (arrowhead) in *lipl32* mRNA-injected larvae (scale bar, 200 μm). In transverse sections, the white arrow indicates colocalization (orange stain) of Myc tag (red) and *wt1b*:GFP fluorescence (green) in pronephric tubules and ducts. Scale bar, 10 μm. (**C**) Cropped western blot shows the expression of LipL32 protein in *lipl32* mRNA-injected larvae at 48 hpf but not in control larvae under the same experimental condition. Whole larva lysates were immunoblotted with a customized antibody against LipL32. The uncropped blot is shown in Supplementary Figure S1. (**D**) Transverse sections (H&E stain, scale bar, 10 μm) show non-fusion of the glomerulus and markedly swelling of the pronephric ducts in *lipl32* mRNA-injected larvae. Quantification of the area of proximal pronephric ducts is shown (*P < 0.05 compared to control). Pt, pronephric tubule; Glm, glomerulus. Circles indicate the location of the pronephric duct. Control larvae were injected with pCS2 (**A**,**B**) or *myc* mRNA (**C**,**D**).

We then sought to examine the pathological role of LipL32 in the developing kidneys by ectopic expression of the protein in zebrafish larvae. To this end, zebrafish embryos were firstly microinjected with a capped RNA of *lipl32* (*myc*-tagged) in the one-cell stage. As shown in Fig. 3A, *in situ* hybridization of *lipl32* mRNA at 24 hpf revealed a widespread expression of LipL32 including the regions of pronephros and the posterior blood island, indicating a successful injection of *lipl32* mRNA. We further confirmed the colocalization of the *lipl32* mRNA expression with *wt1b*:GFP fluorescence in pronephric tubules and ducts, suggesting that the expression of LipL32 may occur in the pronephric kidneys. Similarly, immunostaining for the Myc-tagged epitope in larvae at 48 hpf demonstrated that LipL32 expression can be detected in the pronephros (Fig. 3B). We also found colocalization of Myc and *wt1b*:GFP signals in the pronephric tubules and ducts (Fig. 3B). These results enabled us to examine the specific effects of LipL32 on the pronephros in our model. The ectopic expression of LipL32 was further confirmed by Western blotting with an antibody against LipL32 on the whole larva lysates from the *lipl32* mRNA-injected larvae at 48 hpf (Fig. 3C and Supplementary Figure S1). We next examined whether the ectopic expression of LipL32 induces pronephric duct damages. As shown in Fig. 3D, edematous degeneration and swelling of pronephric epithelium were observed in the *lipl32* mRNA-injected larvae, suggesting that LipL32 may directly contribute to acute tubular injury in leptospiral infection.

### Expression of *lipl32* mRNA impaired kidney development

**Figure 4:**
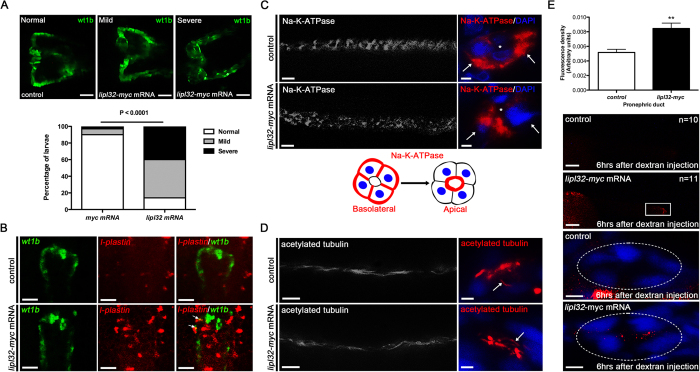
LipL32 induced pronephric malformation, inflammatory cell infiltration and translocation of NA-K-ATPase in zebrafish tubular epithelial cells. (**A**) Quantification of normal, mild or severe deformities of pronephros between groups of *wt1b*:GFP larvae at 48 hpf (n = 30 to 35 in each group). Photos were collected by *in vivo* observation under fluorescence microscopy (dorsal view, anterior to the left). Pronephric kidneys show abnormalities in glomerular fusion, cystic changes, and deformities of pronephric tubules and ducts. Scale bar, 50 μm. (**B**) *In situ* hybridization shows that *l-plastin* positive cells (pseudo-colored by red) were increased in *lipl32* mRNA-injected larvae compared to control. Arrows indicate a colocalization (orange) of GFP staining (green) and *l-plastin* in the pronephric tubule and duct. Scale bar, 50 μm. (**C**) Immunostaining for NA-K-ATPase shows the normal basolateral location of NA-K-ATPase in the pronephric ducts was disorganized in *lipl32* mRNA injected larvae (scale bar, 10 μm). Arrows indicate the basolateral cell surface. Right panels are transverse sections on whole-mount stained larvae on the left (scale bar, 2 μm). A diagram illustrating the changes in the cellular location of NA-K-ATPase is shown. (**D**) Immunostaining for acetyl-tubulin shows no significant differences of pronephric cilia (arrows) between groups (scale bar, left panel, 10 μm, right panel, 2 μm). (**E**) The retention rates of 10-kDa rhodamine dextran as measured from the boxed region of the posterior pronephric ducts 6 hours after pericardial injection were significantly reduced in the *lipl32* mRNA-injected larvae compared to *myc*-mRNA controls (**P < 0.01, n = 10 to 11 in each group, scale bar, 100 μm). Transverse sections demonstrate retention of rhodamine fluorescence in the lumen of pronephric ducts (circle) in *lipl32* mRNA-injected larvae (scale bar, 5 μm).

We next tested whether ectopic expression of *lipl32* mRNA in zebrafish embryo perturbs the development of pronephros. As shown in Fig. 4A, the percentage of pronephric kidney abnormalities (mild and severe) was significantly increased in *lipl32* mRNA-injected larvae compared with control (30/35 vs 3/30, *P* < 0.0001). To investigate the underlying mechanism of kidney injury, we performed *in situ* hybridization for a macrophage marker, *l-plastin.* The expression of *l-plastin* was markedly increased in *lipl32* mRNA-injected larvae and the staining was merged to the *wt1b*:GFP signal (Fig. 4B), suggesting that these inflammatory cells infiltrated the pronephric kidneys. Meanwhile, we tested if LipL32 could affect NA-K-ATPase or cilia in renal epithelial cells. As illustrated in Fig. 4C, the expression of LipL32 disoriented the basolateral location of NA-K-ATPase to the apical membrane of the renal epithelial cells. In contrast, immunostaining for the primary cilia, which is responsible for the mechanosensation of renal tubular epithelium, did not show any structural abnormality in *lipl32* mRNA-injected larvae (Fig. 4D). These results demonstrate that LipL32 may cause pronephric kidney injury by inducing inflammation and mislocalization of NA-K-ATPase. We also found that the glomerular filtration rates, as estimated by the excretion of injected 10-kDa rhodamine dextran, were significantly reduced in the *lipl32* mRNA-injected larvae compared to controls (Fig. 4E), indicating a functional impairment of the affected pronephric kidneys.

### LipL32 triggered pronephric inflammation through the TL2R pathway

**Figure 5:**
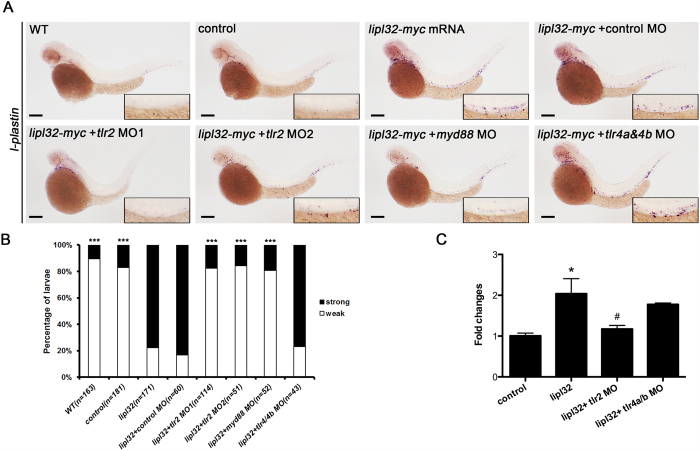
LipL32 promoted pronephric inflammation through the TL2R pathway in zebrafish. Morpholino (MO) knockdown of *tlr2* but not *tlr4a* and *tlr4b* attenuated the expression of *l-plastin* induced by microinjection of *myc*-tagged *lipl32* mRNA. (**A**) *In situ* hybridization for *l-plastin* in zebrafish larvae at 48 hpf, with MO-knockdown of *tlr2*, *myd88* and *tlr4a/4b* in response to *lipl32*-*myc* mRNA injection. Wild-type (WT) larvae and *myc* mRNA-injected control larvae are shown. All panels are lateral view (head toward the left). The insets are enlarged views of the corresponding boxed regions containing the pronephric ducts. (**B**) Comparative frequencies of strong and weak staining of *l-plastin* with different MOs (n = 43 to 171 from two experiments, ***P < 0.0001 versus *lipl32* mRNA). (**C**) Quantitative real-time RT-PCR analyses show that *tlr2* MO inhibited the expression of *l-plastin* induced *by* LipL32. Results shown are the mean ± SEM from three independent experiments carried out in duplicate (*P < 0.05 versus controls, ^#^*P* < 0.05 versus LipL32). Scale bar, 200 μm.

We then investigated whether LipL32 triggers inflammation through the TLR2 pathway according to our previous *in vitro* studies^19^,^20^,^32^. As shown in Fig. 5A,B, the ectopic expression of *lipl32* mRNA triggers the accumulation of *l-plastin* positive cells in the posterior blood island and the region surrounding pronephric ducts. The inflammatory response was significantly abolished (*P* < 0.0001) in the *tlr-2* knockdown larvae but remained unchanged with concomitant *tlr4a* and *tlr4b* knockdown. Knockdown of the *tlr2* downstream transducers myeloid differentiation factor 88 (*myd88*) also blocked (*P* < 0.0001) the *lipl32*-mediated *l-plastin* expression. Quantitative real-time RT-PCR confirmed that the expression of *l-plastin* mRNA was reduced significantly after knocking down *tlr2* (*P* < 0.05) rather than *tlr4a and tlr4b* (Fig. 5C).

### Injection of LipL32 protein through the tail vein

**Figure 6:**
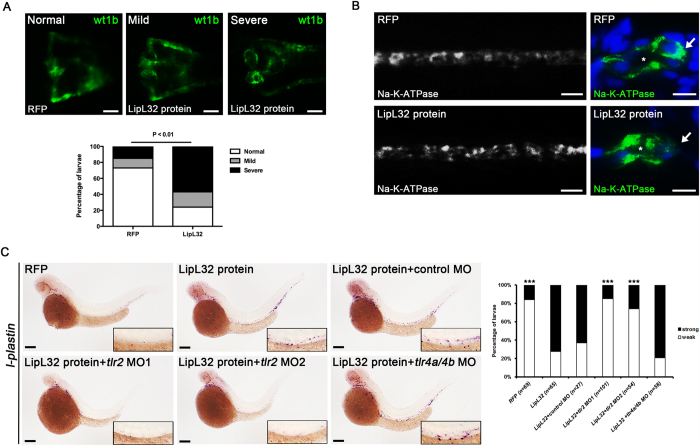
Injection of recombinant LipL32 protein induced pronephric kidney deformity, inflammation, and translocation of NA-K-ATPase. (**A**) The frequencies of deformities in pronephros were significantly increased in the LipL32-injected group compared to the control group (*wt1b*:GFP larvae at 48 hpf, n = 21 to 26 in each group, *P* < 0.01). Purified recombinant LipL32 protein was microinjected into the tail vein of zebrafish larvae at 24 hpf. Control larvae were injected with RFP. Photos were collected by *in vivo* observation under fluorescence microscopy (dorsal view, anterior to the left). Scale bar, 50 μm. (**B**) Immunostaining for NA-K-ATPase shows disruption of the basolateral location of NA-K-ATPase in the pronephric ducts in LipL32-injected larvae at 48 hpf. Right panels (scale bar, 5 μm) are the transverse sections of whole-mount stained larvae on the left (scale bar, 20 μm). Asterisks denote the lumens of pronephric ducts. Arrows indicate the basolateral cell surface. (**C**) *In situ* hybridization at 48 hpf shows that *l-plastin*-positive cells were increased after injection of LipL32 and the response was blocked by morpholino knockdown of *tlr2* but not *tl4a* and *tl4b*. The insets are enlarged views of the corresponding regions of pronephric ducts. Scale bar, 200 μm. The diagram indicates the frequencies of strong and weak staining of *l-plastin* with different MOs (n = 27 to 101 from three experiments, ***P < 0.0001 versus LipL32 protein).

We finally investigated the virulence of LipL32 protein by direct injection of a recombinant LipL32 protein into the tail vein of zebrafish larvae at 24 hpf. Successful injection of the recombinant protein to the circulation was confirmed by the presence of co-injected rhodamine fluorescence. As shown in Fig. 6A, the frequency of pronephric kidney deformity was significantly increased compared to control larvae (16/21 vs 7/26, *P* < 0.01). Injection of LipL32 protein also disrupted the expression of NA-K-ATPase, which was partially translocated from the basolateral membrane to the apical cell membrane (Fig. 6B). Furthermore, the expression of *l-plastin* mRNA in the pronephric area as shown by *in situ* hybridization was markedly increased after injection of LipL32 and was significantly suppressed (*P* < 0.0001) with morpholino knockdown of *tlr2* but not of *tlr4a* and *tlr4b* (Fig. 6C). These data confirmed that circulating LipL32 can induce inflammation and kidney malformation in zebrafish larvae.

### TLR2 blockade ameliorated the kidney injury induced by LipL32

**Figure 7:**
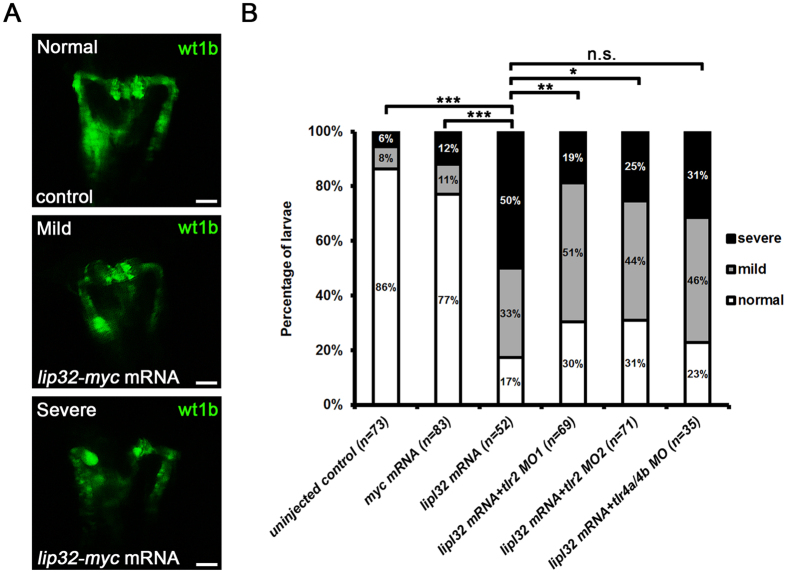
TLR2 blockade ameliorated the kidney injury induced by LipL32. (**A**) Pronephric kidney injuries were induced in the *lipl32* mRNA-injected *wt1b*:GFP larvae at 48 hpf. Representative images of normal, mild and severe deformities in pronephros are shown. Morphological deformities were assessed according to the criteria described in Methods. Photos were collected under fluorescence microscopy (dorsal view, anterior to the top). Scale bar, 50 μm. (**B**) Morpholino (MO) knockdown of *tlr2* but not *tlr4a* and *tlr4b* significantly attenuated the LipL32-induced kidney injuries. The diagram indicates the frequencies of normal, mild and severe deformities in the *wt1b:*GFP larvae microinjected with different TLR MOs (n = 32 to 83 from three experiments, *P < 0.05, **P < 0.01, ***P < 0.001 versus *lipl32* mRNA, n.s., not significant).

We further determined the potential rescue effects of TLR blockade on the LipL32-induced injury to the pronephros. As shown in Fig. 7, the frequencies of kidney deformities caused by the ectopic expression of *lipl32* mRNA in *wt1b:*GFP larvae were significantly reduced by microinjection of *tlr2* MOs (*P* < 0.05) but not *tlr4a* and *tlr*4*b* MOs (*P* = 0.2275). These results suggest that LipL32 might induce kidney injury predominantly through a TLR2-dependent pathway in zebrafish larvae.

## Discussion

Here we used a zebrafish model for investigating the potential virulence mechanisms of LipL32 in leptospirosis. We found that *L.* Shermani infection induced pronephric kidney injury in zebrafish larvae. Meanwhile, ectopic expression of LipL32 in zebrafish larvae prompted macrophage infiltration to the pronephric kidneys through the innate immunity TLR2 pathway, disrupted the basolateral NA-K-ATPase location, and impaired the normal kidney structure. These results support the use of zebrafish as a model organism for leptospiral infection^30^.

Zebrafish has been used as a model organism for various diseases and may provide a unique opportunity to observe early pathological changes. Zebrafish has a fully functional innate immune system by 32 hpf with the development of circulating systems and phagocytes^30^. The pattern-recognition receptor system in zebrafish is highly similar to that in other vertebrates, which makes it a useful tool for examining host-microbe interactions^33^,^34^. An earlier study that injected *L. interrogans* into the caudal vein of zebrafish larvae has shown that leptospires are readily ingested by macrophages, which are later found specifically accumulated in the ventral wall of the dorsal aorta, equivalent to the aorta-gonad-mesonephros (AGM) in mammals^30^. In this current study, we further demonstrated that overexpression of LipL32 induced accumulation of macrophages in the posterior blood island and the pronephros, suggesting that LipL32 may participate in guiding macrophages to the target organs. Thus, LipL32 might be a potential leptospiral adhesin that is important in the specific interactions to host substrates^35^.

LipL32 is a major immunogen during leptospiral infection in humans and mammals^9^. In hamster model of leptospirosis, immunohistochemical analyses of *L.* kirschneri–infected kidneys have demonstrated the intense LipL32 expression in proximal tubule cells and the interstitium^16^. Vaccination with LipL32 reduces the severity of kidney inflammation in the hamsters infected with *L. interrogans* serovar Canicola in hamsters^36^. In this study, we demonstrated that the ectopic expression of LipL32 directly induced an inflammatory reaction and caused kidney injury, suggesting that LipL32 is a virulence factor and plays an important role in mediating the pathogenesis of renal injury in leptospirosis^37^.

Disturbances of renal electrolyte handling may occur in acute leptospirosis. This could lead to decreased renal water absorption and renal salt losing commonly seen as non-oliguric renal failure in leptospirosis^10^. Previous studies have demonstrated that the expression and function of NA-K-ATPase are inhibited by L. *interrogans* glycoproteins^38^,^39^,^40^. The addition of GLP extracted from *L.* interrogans specifically inhibits the Na-K-ATPase in rabbit kidney epithelial cells^39^. Downregulation of NA-K-ATPase and other ion transporters have been found in L. *interrogans*-infected mice^41^. Basolaterally located Na-K-ATPase is essential for transfer of salt and water across epithelium to interstitium and its dislocation has been related to alveolar edema in leptospirosis^11^. Our data are consistent with these findings and provide *in vivo* evidence that LipL32 also perturbed the expression of NA-K-ATPase in renal epithelial cells. Whether LipL32 might interact with NA-K-ATPase directly warrants future studies.

The TLR pathways have been shown to play a pivotal role in the pathogenesis of leptospirosis^42^. It has been demonstrated that TLR2-deficient mice showed a reduced cytokine response to leptospiral LPS challenge, and LPS activates macrophages through TLR2 in cultured monocytes^9^. Our previous studies have shown that LipL32 activates TLR2 and downstream genes in cultured mouse medullary ascending limb cells and proximal tubule cells^19^,^43^. Experimental evidence from an atomic force microscope study suggests that LipL32 binds directly to TLR2, but not TLR4, on the renal cell surface^32^. In the current study, we further demonstrated that LipL32 induced leukocyte infiltration through the TLR2 pathway *in vivo.* These finding strongly support that LipL32 binds directly to TLR2 to mediate its virulence functions. Future research could also explore the potential LipL32 ligands other than immune receptors^44^,^45^.

Our results are different to a previous study which showed that a LipL32 mutant constructed in *L. interrogans* was still able to infect hamsters and rats^46^. This discrepancy may be explained partly by a high degree of redundancy in leptospiral proteins involved in adhesion, survival, and renal colonization^47^. However, by direct expression of LipL32 in the zebrafish model, our data demonstrated clearly the virulence of LipL32 in causing host inflammation and target organ damage. It is also worth noting that other outer membrane proteins such as Loa22, OmpL1, p31/LipL45, and LenA have been regarded as potential leptospiral adhesins and might warrant further investigation^35^,^48^.

In conclusion, we have shown that LipL32 promotes inflammation and induces kidney injury in zebrafish larvae. This investigation supports the hypothesis that LipL32 contributes to the pathogenesis of renal injury in leptospirosis. Our results support the use of zebrafish as a model organism for studying the disease mechanism of leptospiral infection. This model might permit the future exploration of the virulence and molecular pathways of different leptospiral outer membrane proteins.

## Methods

### Maintenance of fish

The study was approved by the Institutional Animal Care and Use Committee of Chang Gung Memorial Hospital and Chang Gung University. The study conformed to the Guide for the Care and Use of Laboratory Animals published by the National Institute of Health. Zebrafish were maintained by standard protocols^49^. The transgenic line *wt1b*:GFP with pronephros specific GFP expression was kindly provided by Prof. Christoph Englert (Fritz Lipmann Institute, Jena, Germany)^50^. Zebrafish embryos were staged according to hours post fertilization (hpf)^51^.

### Bacterial strains, growth conditions, and zebrafish larva infection

*L. santarosai* serovar Shermani strain LT821 (ATCC number 43286; a pathogenic strain) and *L. biflexa* serovar Patoc (ATCC number 23582; a non-pathogenic strain) were purchased from the American Type Culture Collection (Manassas, VA) and propagated at 28 °C under aerobic conditions in medium containing 10% *Leptospira* Enrichment Ellinghausen-McCullough-Johnson-Harris (EMJH) and 90% *Leptospira* Medium Base EMJH (Difco, BD Diagnostics, Sparks, MD). Bacterial densities were counted with a CASY-Model TT cell counter and analyzer (Roche Innovatis AG, Casy-Technology, Reutlingen, Germany). For zebrafish infection, living 24 hpf larvae were manually dechorionated and distributed in 6-well plates (15–30 larvae per well), immersed in E3 media containing leptospires at a concentration of 1 × 10^6^ colony-forming units (CFU)/ml, and maintained at 28 °C in an incubator at the RG2 laboratory.

### Microinjection of *lipl32* mRNA

An *myc* epitope-tagged pCS2-MT-LipL32 plasmid was generated by cloning *lipl32* cDNA (798 bp, amino acids 1–266) from pathogenic *L. santarosai* serovar Shermani genomic DNA, into the Cla1/EcoR1 sites of the pCS2-MT plasmid (Promega). The *lipl32* sequence was confirmed by DNA sequencing. *In vitro* transcribed capped RNA of *lipl32* (*myc*-tagged, 600 ng/μl, 4 nl) was microinjected in the one-cell stage of zebrafish embryos according to standard protocols^52^.

### Microinjection of LipL32 protein

Detoxified LipL32 protein (amino acids 21–272) was prepared as described previously^20^,^32^. Larvae were anesthetized using 0.02% tricaine. LipL32 recombinant protein (0.07mg/ml, 4 nl) was injected into the caudal vein at 24 hpf together with a 10-kDa rhodamine–dextran (Invitrogen) to confirm the injection into blood circulation. Control larvae were injected with phosphate-buffered saline (PBS) or red fluorescent protein (RFP).

### Morpholino knockdown

Antisense morpholino oligonucleotides (MO) against zebrafish *tlr2, tlr4a, tlr4b*, and *myd88* were designed and synthesized by Gene-Tools (Philomath, OR)^53^,^54^. One- to two-cell stage embryos were microinjected with 0.125 mM antisense MOs. The sequences of MO used in this study are as follows: *tlr2* MO1 (translating blocking), 5′-CCTGACTGCCATTATTGTGTCTACT-3′, *tlr2* MO2 (translating blocking) 5′-AGTCATTGTTCCTACGAGTCTCATC-3′^53^, *tlr4a* MO (splice blocking), 5′-GTAATGGCATTACTTACCTTGACAG-3′^54^, *tlr4b* MO (splice blocking), 5′-CTATGTAATGTTCTTACCTCGGTAC-3′^54^, *myd88* MO (translating blocking) 5′-TACCATAACCTGTGTTATCGAGGGA-3′^54^, and standard control MO, 5′-CCTCTTACCTCAGTTACAATTTATA-3′.

### Morphological abnormalities in pronephros

Pronephros was observed in living transgenic *wt1b*:GFP zebrafish larvae under fluorescence microscopy at 48 hpf 55. Developmental abnormalities of the pronephros including glomerular malformation and tubular dilatation were categorized as normal, mild (glomerular malformation or a loss of tubular angle) or severe deformity (glomerular malformation and a loss of tubular angle)^50^,^56^,^57^.

### Histology analysis

Zebrafish larvae were fixed in 4% paraformaldehyde in PBS overnight at 4 °C, embedded in glycol methacrylate (JB-4; Polyscience), and cut at 5 μm. Slides were stained with Hematoxylin and Eosin (H&E). Quantitative image analysis for the area of pronephric ducts was performed using ImageJ software.

### Immunohistochemistry

Immunostaining was performed in whole-mount larvae as described previously^58^. A mouse anti-NA-K-ATPase alpha-subunit (a5) antibody (1:200, Developmental Studies Hybridoma Bank) was used to label the location of NA-K-ATPase in the pronephric epithelial cells. A mouse anti-acetylated alpha-tubulin antibody (1:200, Sigma) was used to label the primary cilia of pronephric ducts. A mouse anti-c-myc (9E10) antibody (1:500, Covance) was used to label the Myc-tag of LipL32 protein expression. A rabbit anti-GFP antibody (1:500, Invitrogen) was used to label the pronephric GFP expression in the *wt1b*:GFP larvae. The secondary antibodies used were Alexa Fluor 488- or 594- conjugated goat anti-mouse IgG and goat anti-rabbit IgG (1:500, Invitrogen). The immunostained larvae were then embedded in OTC medium and cryosectioned. Sections were mounted in Vectashield (Vector Laboratories) with DAPI and observed under fluorescence microscopy.

### Rhodamine-dextran injection

For renal function estimation, a 10-kDa rhodamine–dextran (Invitrogen) was injected into the pericardium of zebrafish larvae at 48 hpf after anesthesia with tricaine^59^. Images of larvae were collected under fluorescence microscopy 6 hours after injection. A region of 200 × 100 pixels containing the pronephric duct and cloaca was selected and the fluorescence intensity was measured using ImageJ software.

### Whole-mount *in situ* hybridization

Larvae were fixed overnight in 4% paraformaldehyde at 4 °C. Whole-mount *in situ* hybridization was performed according to published protocols^49^. Antisense digoxigenin-labeled probes were made for *lipl32* and *l-plastin* from corresponding cDNA constructs.

### RT-PCR

The expression of *lipl32* and *flaB* mRNAs was examined by RT-PCR to confirm the infection of pathogenic *Leptospira* species according to the previously described methods^31^,^60^. Larvae were mixed and ground in liquid nitrogen for total RNA extraction using TRIzol Reagent (Life Technologies). We obtained cDNA from the total RNA using the First Strand cDNA synthesis kit for RT-PCR (AMV) (Roche Diagnostics) following the manufacturer’s instructions. The primers used were: *lipl32*, forward 5′-CGCTGAAATGGGAGTTCGTATGATT-3′ and reverse 5′- CCAACAGATGCAACGAAAGATCCTTT-3′^60^; *flaB*, forward 5′- CTCACCGTTCTCTAAAGTTCAAC-3′ and reverse 5′- TGAATTCGGTTTCATATTTGCC-3′^31^. Zebrafish *ß-actin* was used as internal control.

### Real-time RT-PCR

Real-time quantitative RT-PCR was performed with the ABI PRISM 7700 sequence detection system (Applied Biosystems) in the presence of SYBR Green. The primers used to amplify zebrafish *l-plastin* were: forward 5′-CCGCTACGACCTGCTGAAG-3′, reverse: 5′-GCGCCATCGAGATTGCAT-3′. The zebrafish ribosomal protein S18 (*rps18)* was used as internal control. The reaction was performed in duplicate for each sample.

### Western blot analysis

Proteins extracted from whole larvae were analyzed by Western blotting following the standard method^49^. Antibodies used include anti-tubulin (1:8000, clone DM1a, Sigma) and a purified custom-made rabbit polyclonal antibody (1:10000) raised against the LipL32 protein (amino acids 21–272).

### Statistical analysis

Continuous variables are expressed as mean ± SEM and compared by using Student’s t-test or one-way ANOVA followed by Newman-Keuls Test. Categorical variables were analyzed using the Fisher’s exact or Chi-square test. Cumulative survival curves were generated by the Kaplan–Meier method and compared with the log-rank test. The standard error of the survival curves was calculated using the Greenwood method. *P-*values < 0.05 are considered statistically significant. All analyses were performed using the Graphpad Prism 5.1 (Graphpad, La Jolla, CA).

## Additional Information

**How to cite this article**: Chang, M.-Y. *et al.* Leptospiral outer membrane protein LipL32 induces inflammation and kidney injury in zebrafish larvae. *Sci. Rep.* **6**, 27838; doi: 10.1038/srep27838 (2016).

## Supplementary Material

Supplementary Fig. S1

## References

[b1] Evangelista K. V. & Coburn J. Leptospira as an emerging pathogen: a review of its biology, pathogenesis and host immune responses. Future Microbiol 5, 1413–1425 (2010). 20860485 10.2217/fmb.10.102 PMC3037011

[b2] Chou L. F. *et al.* Sequence of Leptospira santarosai serovar Shermani genome and prediction of virulence-associated genes. Gene 511, 364–370 (2012). 23041083 10.1016/j.gene.2012.09.074

[b3] Yang C. W. *et al.* Leptospirosis: an ignored cause of acute renal failure in Taiwan. Am J Kidney Dis 30, 840–845 (1997). 9398130 10.1016/s0272-6386(97)90091-3

[b4] Slamti L., de Pedro M. A., Guichet E. & Picardeau M. Deciphering morphological determinants of the helix-shaped Leptospira. J Bacteriol 193, 6266–6275 (2011). 21926230 10.1128/JB.05695-11 PMC3209227

[b5] Mgode G. F., Mhamphi G. G., Katkweba A. S. & Thomas M. Leptospira infections in freshwater fish in Morogoro Tanzania: a hidden public health threat. Tanzan J Health Res 16, 112–117 (2014). 26875305 10.4314/thrb.v16i2.7

[b6] Yang C. W. *et al.* The Leptospira outer membrane protein LipL32 induces tubulointerstitial nephritis-mediated gene expression in mouse proximal tubule cells. J Am Soc Nephrol 13, 2037–2045 (2002). 12138134 10.1097/01.asn.0000022007.91733.62

[b7] Haake D. A. & Levett P. N. Leptospirosis in humans. Curr Top Microbiol Immunol 387, 65–97 (2015). 25388133 10.1007/978-3-662-45059-8_5 PMC4442676

[b8] Spichler A., Athanazio D. A., Furtado J., Seguro A. & Vinetz J. M. Case report: severe, symptomatic hypomagnesemia in acute leptospirosis. Am J Trop Med Hyg 79, 915–917 (2008). 19052304 PMC2637037

[b9] Werts C. *et al.* Leptospiral lipopolysaccharide activates cells through a TLR2-dependent mechanism. Nat Immunol 2, 346–352 (2001). 11276206 10.1038/86354

[b10] Cesar K. R. *et al.* Renal involvement in leptospirosis: the effect of glycolipoprotein on renal water absorption. PLoS One 7, e37625 (2012). 22701573 10.1371/journal.pone.0037625 PMC3368910

[b11] Goncalves-de-Albuquerque C. F. *et al.* Murine lung injury caused by Leptospira interrogans glycolipoprotein, a specific Na/K-ATPase inhibitor. Respir Res 15, 93 (2014). 25265888 10.1186/s12931-014-0093-2 PMC4151191

[b12] Wang H. *et al.* Leptospiral hemolysins induce proinflammatory cytokines through Toll-like receptor 2-and 4-mediated JNK and NF-kappaB signaling pathways. PLoS One 7, e42266 (2012). 22870312 10.1371/journal.pone.0042266 PMC3411626

[b13] Evangelista K. V. *et al.* Identification of cell-binding adhesins of Leptospira interrogans. PLoS Negl Trop Dis 8, e3215 (2014). 25275630 10.1371/journal.pntd.0003215 PMC4183468

[b14] Cullen P. A., Cordwell S. J., Bulach D. M., Haake D. A. & Adler B. Global analysis of outer membrane proteins from Leptospira interrogans serovar Lai. Infect Immun 70, 2311–2318 (2002). 11953365 10.1128/IAI.70.5.2311-2318.2002 PMC127947

[b15] Haake D. A. & Zuckert W. R. The leptospiral outer membrane. Curr Top Microbiol Immunol 387, 187–221 (2015). 25388136 10.1007/978-3-662-45059-8_8 PMC4419373

[b16] Haake D. A. *et al.* The leptospiral major outer membrane protein LipL32 is a lipoprotein expressed during mammalian infection. Infect Immun 68, 2276–2285 (2000). 10722630 10.1128/iai.68.4.2276-2285.2000 PMC97414

[b17] Guerreiro H. *et al.* Leptospiral proteins recognized during the humoral immune response to leptospirosis in humans. Infect Immun 69, 4958–4968 (2001). 11447174 10.1128/IAI.69.8.4958-4968.2001 PMC98588

[b18] Tian Y. C. *et al.* Leptospiral outer membrane protein induces extracellular matrix accumulation through a TGF-beta1/Smad-dependent pathway. J Am Soc Nephrol 17, 2792–2798 (2006). 16928805 10.1681/ASN.2006020159

[b19] Yang C. W. *et al.* Toll-like receptor 2 mediates early inflammation by leptospiral outer membrane proteins in proximal tubule cells. Kidney Int 69, 815–822 (2006). 16437059 10.1038/sj.ki.5000119

[b20] Lo Y. Y. *et al.* Essential calcium-binding cluster of Leptospira LipL32 protein for inflammatory responses through the Toll-like receptor 2 pathway. J Biol Chem 288, 12335–12344 (2013). 23486465 10.1074/jbc.M112.418699 PMC3636917

[b21] Vivian J. P. *et al.* Crystal structure of LipL32, the most abundant surface protein of pathogenic Leptospira spp. J Mol Biol 387, 1229–1238 (2009). 19236879 10.1016/j.jmb.2009.02.038

[b22] Tung J. Y., Yang C. W., Chou S. W., Lin C. C. & Sun Y. J. Calcium binds to LipL32, a lipoprotein from pathogenic Leptospira, and modulates fibronectin binding. J Biol Chem 285, 3245–3252 (2010). 19948735 10.1074/jbc.M109.006320 PMC2823465

[b23] Hoke D. E., Egan S., Cullen P. A. & Adler B. LipL32 is an extracellular matrix-interacting protein of Leptospira spp. and Pseudoalteromonas tunicata. Infect Immun 76, 2063–2069 (2008). 18285490 10.1128/IAI.01643-07 PMC2346718

[b24] Chaemchuen S., Rungpragayphan S., Poovorawan Y. & Patarakul K. Identification of candidate host proteins that interact with LipL32, the major outer membrane protein of pathogenic Leptospira, by random phage display peptide library. Vet Microbiol 153, 178–185 (2011). 21592685 10.1016/j.vetmic.2011.04.030

[b25] Stockhammer O. W., Zakrzewska A., Hegedus Z., Spaink H. P. & Meijer A. H. Transcriptome profiling and functional analyses of the zebrafish embryonic innate immune response to Salmonella infection. J Immunol 182, 5641–5653 (2009). 19380811 10.4049/jimmunol.0900082

[b26] Chao C. C. *et al.* Zebrafish as a model host for Candida albicans infection. Infect Immun 78, 2512–2521 (2010). 20308295 10.1128/IAI.01293-09 PMC2876552

[b27] Neely M. N., Pfeifer J. D. & Caparon M. Streptococcus-zebrafish model of bacterial pathogenesis. Infect Immun 70, 3904–3914 (2002). 12065534 10.1128/IAI.70.7.3904-3914.2002 PMC128100

[b28] Chu H. & Mazmanian S. K. Innate immune recognition of the microbiota promotes host-microbial symbiosis. Nat Immunol 14, 668–675 (2013). 23778794 10.1038/ni.2635 PMC4109969

[b29] Wingert R. A. & Davidson A. J. The zebrafish pronephros: a model to study nephron segmentation. Kidney Int 73, 1120–1127 (2008). 18322540 10.1038/ki.2008.37

[b30] Davis J. M., Haake D. A. & Ramakrishnan L. Leptospira interrogans stably infects zebrafish embryos, altering phagocyte behavior and homing to specific tissues. PLoS Negl Trop Dis 3, e463 (2009). 19547748 10.1371/journal.pntd.0000463 PMC2693671

[b31] Natarajaseenivasan K. *et al.* FlaB PCR-based identification of pathogenic leptospiral isolates. J Microbiol Immunol Infect 43, 62–69 (2010). 20434125 10.1016/S1684-1182(10)60009-6

[b32] Hsu S. H. *et al.* Leptospiral outer membrane lipoprotein LipL32 binding on toll-like receptor 2 of renal cells as determined with an atomic force microscope. Biochemistry 49, 5408–5417 (2010). 20513152 10.1021/bi100058w

[b33] van der Sar A. M., Appelmelk B. J., Vandenbroucke-Grauls C. M. & Bitter W. A star with stripes: zebrafish as an infection model. Trends Microbiol 12, 451–457 (2004). 15381194 10.1016/j.tim.2004.08.001

[b34] Stein C., Caccamo M., Laird G. & Leptin M. Conservation and divergence of gene families encoding components of innate immune response systems in zebrafish. Genome Biol 8, R251 (2007). 18039395 10.1186/gb-2007-8-11-r251 PMC2258186

[b35] Robbins G. T. *et al.* Evaluation of cell binding activities of Leptospira ECM adhesins. PLoS Negl Trop Dis 9, e0003712 (2015). 25875373 10.1371/journal.pntd.0003712 PMC4397020

[b36] Humphryes P. C. *et al.* Vaccination with leptospiral outer membrane lipoprotein LipL32 reduces kidney invasion of Leptospira interrogans serovar canicola in hamsters. Clin Vaccine Immunol 21, 546–551 (2014). 24521782 10.1128/CVI.00719-13 PMC3993109

[b37] Rajapakse S., Rodrigo C., Handunnetti S. M. & Fernando S. D. Current immunological and molecular tools for leptospirosis: diagnostics, vaccine design, and biomarkers for predicting severity. Ann Clin Microbiol Antimicrob 14, 2 (2015). 25591623 10.1186/s12941-014-0060-2 PMC4299796

[b38] Burth P., YounesIbrahim M., Goncalez F. H. F. S., Costa E. R. & Faria M. V. C. Purification and characterization of a Na+, K+ ATPase inhibitor found in an endotoxin of Leptospira interrogans. Infect Immun 65, 1557–1560 (1997). 9119504 10.1128/iai.65.4.1557-1560.1997 PMC175170

[b39] Younes-Ibrahim M. *et al.* Inhibition of Na,K-ATPase by an endotoxin extracted from Leptospira interrogans: a possible mechanism for the physiopathology of leptospirosis. C R Acad Sci III 318, 619–625 (1995). 7671008

[b40] Younes-Ibrahim M. *et al.* Na, K-ATPase: a molecular target for Leptospira interrogans endotoxin. Braz J Med Biol Res 30, 213–223 (1997). 9239307 10.1590/s0100-879x1997000200009

[b41] Lacroix-Lamande S. *et al.* Downregulation of the Na/K-ATPase pump by leptospiral glycolipoprotein activates the NLRP3 inflammasome. J Immunol 188, 2805–2814 (2012). 22323544 10.4049/jimmunol.1101987

[b42] Ko A. I., Goarant C. & Picardeau M. Leptospira: the dawn of the molecular genetics era for an emerging zoonotic pathogen. Nat Rev Microbiol 7, 736–747 (2009). 19756012 10.1038/nrmicro2208 PMC3384523

[b43] Yang C. W. *et al.* Leptospira outer membrane protein activates NF-kappaB and downstream genes expressed in medullary thick ascending limb cells. J Am Soc Nephrol 11, 2017–2026 (2000). 11053477 10.1681/ASN.V11112017

[b44] Werts C. Leptospirosis: a Toll road from B lymphocytes. Chang Gung Med J 33, 591–601 (2010). 21199604

[b45] Fanton d’Andon M. *et al.* Leptospira Interrogans induces fibrosis in the mouse kidney through Inos-dependent, TLR- and NLR-independent signaling pathways. PLoS Negl Trop Dis 8, e2664 (2014). 24498450 10.1371/journal.pntd.0002664 PMC3907306

[b46] Murray G. L. *et al.* Major surface protein LipL32 is not required for either acute or chronic infection with Leptospira interrogans. Infect Immun 77, 952–958 (2009). 19103763 10.1128/IAI.01370-08 PMC2643616

[b47] Adler B. & de la Pena Moctezuma A. Leptospira and leptospirosis. Vet Microbiol 140, 287–296 (2010). 19345023 10.1016/j.vetmic.2009.03.012

[b48] Lin X., Sun A., Ruan P., Zhang Z. & Yan J. Characterization of conserved combined T and B cell epitopes in Leptospira interrogans major outer membrane proteins OmpL1 and LipL41. BMC Microbiol 11, 21 (2011). 21269437 10.1186/1471-2180-11-21 PMC3038132

[b49] Westerfield M. The zebrafish book. A guide for the laboratory use of zebrafish (Danio rerio), 4th ed. University of Oregon Press, Eugene, OR (2000).

[b50] Perner B., Englert C. & Bollig F. The Wilms tumor genes wt1a and wt1b control different steps during formation of the zebrafish pronephros. Dev Biol 309, 87–96 (2007). 17651719 10.1016/j.ydbio.2007.06.022

[b51] Kimmel C. B., Ballard W. W., Kimmel S. R., Ullmann B. & Schilling T. F. Stages of embryonic development of the zebrafish. Dev Dyn 203, 253–310 (1995). 8589427 10.1002/aja.1002030302

[b52] Yuan S. & Sun Z. Microinjection of mRNA and morpholino antisense oligonucleotides in zebrafish embryos. J Vis Exp 27, e1113 (2009). 10.3791/1113 PMC2762915 19488022

[b53] Yang S., Marin-Juez R., Meijer A. H. & Spaink H. P. Common and specific downstream signaling targets controlled by Tlr2 and Tlr5 innate immune signaling in zebrafish. BMC Genomics 16, 547 (2015). 26208853 10.1186/s12864-015-1740-9 PMC4514945

[b54] Sepulcre M. P. *et al.* Evolution of lipopolysaccharide (LPS) recognition and signaling: fish TLR4 does not recognize LPS and negatively regulates NF-kappaB activation. J Immunol 182, 1836–1845 (2009). 19201835 10.4049/jimmunol.0801755

[b55] Chang M. Y. *et al.* Inhibition of the P2X7 receptor reduces cystogenesis in PKD. J Am Soc Nephrol 22, 1696–1706 (2011). 21636640 10.1681/ASN.2010070728 PMC3171940

[b56] Peng H. C. *et al.* Nephrotoxicity assessments of acetaminophen during zebrafish embryogenesis. Comp Biochem Physiol C Toxicol Pharmacol 151, 480–486 (2010). 20170747 10.1016/j.cbpc.2010.02.004

[b57] Westhoff J. H. *et al.* Development of an automated imaging pipeline for the analysis of the zebrafish larval kidney. PLoS One 8, e82137 (2013). 24324758 10.1371/journal.pone.0082137 PMC3852951

[b58] Krens S. F. *et al.* Distinct functions for ERK1 and ERK2 in cell migration processes during zebrafish gastrulation. Dev Biol 319, 370–383 (2008). 18514184 10.1016/j.ydbio.2008.04.032

[b59] Hentschel D. M. *et al.* Acute renal failure in zebrafish: a novel system to study a complex disease. Am J Physiol Renal Physiol 288, F923–929 (2005). 15625083 10.1152/ajprenal.00386.2004

[b60] Levett P. N. *et al.* Detection of pathogenic leptospires by real-time quantitative PCR. J Med Microbiol 54, 45–49 (2005). 15591254 10.1099/jmm.0.45860-0

