# Supplementary material for: Leptospiral outer membrane protein LipL32 induces inflammation and kidney injury in zebrafish larvae
Source: Sci Rep. 2016 Jun 9;6:27838. doi: 10.1038/srep27838 (PMC4899798; doi:10.1038/srep27838)
Supplement: Supplementary Fig. S1 [file srep27838-s1.pdf]

Supplementary information

Leptospiral outer membrane protein LipL32 induces inflammation and kidney injury in zebrafish larvae

Ming-Yang Chang, Yi-Chuan Cheng, Shen-Hsing Hsu, Tsu-Lin Ma, Li-Fang Chou, Hsiang-Hao Hsu, Ya-Chung Tian, Yung-Chang Chen, Yuh-Ju Sun, Cheng-Chieh Hung, Rong-Long Pan, Chih-Wei Yang.

Supplementary Figures

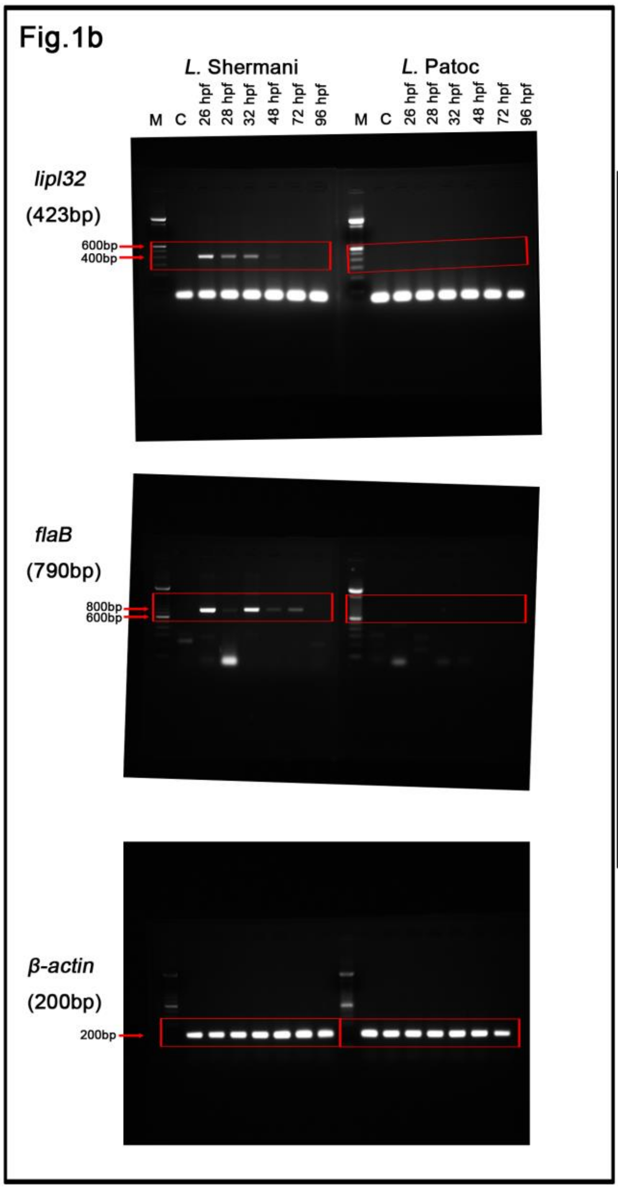

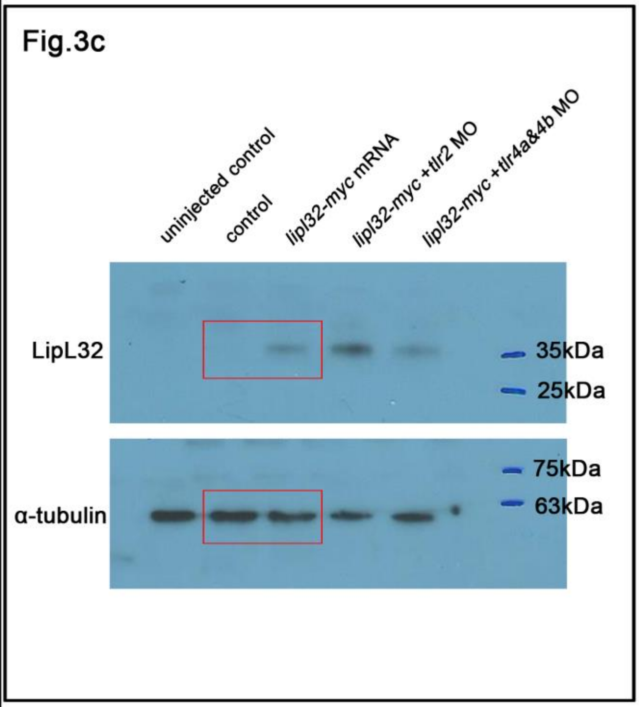

Supplementary Fig. S1. Uncropped gels and blots corresponding to the cropped images reported in Fig.1b and Fig.3c.
